# Don’t Demean “Invasives”: Conservation and Wrongful Species Discrimination

**DOI:** 10.3390/ani9110871

**Published:** 2019-10-27

**Authors:** C.E. Abbate, Bob Fischer

**Affiliations:** 1 Philosophy Department, University of Nevada, Las Vegas, NV 89154, USA; 2 Philosophy Department, Texas State University, San Marcos, TX 78666, USA

**Keywords:** invasive species, discrimination, animal rights

## Abstract

**Simple Summary:**

According to the United States Department of Agriculture (USDA), “invasive” species are “alien” or non-native species “whose introduction does or is likely to cause economic or environmental harm or harm to human health”. On this basis, many state agencies encourage the eradication of invasive species. We argue that this constitutes wrongful discrimination against members of endangered species. Although there may well be cases where it is permissible to kill them. the reasons that these agencies provide often are not sufficient to justify the killing. More importantly for our purposes, though, the killing is not the only injustice here. Additionally, it is wrong to categorize animals as invasive, and we show how to reach this conclusion by borrowing from Deborah Hellman’s account of wrongful discrimination.

**Abstract:**

It is common for conservationists to refer to non-native species that have undesirable impacts on humans as “invasive”. We argue that the classification of any species as “invasive” constitutes wrongful discrimination. Moreover, we argue that its being wrong to categorize a species as invasive is perfectly compatible with it being morally permissible to kill animals—assuming that conservationists “kill equally”. It simply is not compatible with the double standard that conservationists tend to employ in their decisions about who lives and who dies.

## 1. Introduction

In the summer of 2019, the Florida Fish and Wildlife Conservation Commission (FWC) updated its webpage on the “invasive green iguana”. Green iguanas are native to Central America, but starting in the 1960s, people began to notice them roaming wild. Although the timeline is unclear, it seems that they were brought into the US as pets. Some were released into the wild by their owners; others probably escaped. Since then, they have flourished in Florida with especially large populations in the southern part of the state. Richard Engeman, a biologist for the National Wildlife Research Center, said in 2018:

“There’s no real way to come up with a valid estimate of the number of green iguanas in Florida. But the number would be gigantic. You could put any number of zeros behind a number, and I would believe it”.

[1]

This has led to predictable conflicts. According to the FWC:

“Green iguanas cause damage to residential and commercial landscape vegetation and are often considered a nuisance by property owners… Some green iguanas cause damage to infrastructure by digging burrows that erode and collapse sidewalks, foundations, seawalls, berms and canal banks. Green iguanas may also leave droppings on docks, moored boats, seawalls, porches, decks, pool platforms, and inside swimming pools. Although primarily herbivores, researchers found the remains of tree snails in the stomachs of green iguanas in Bill Baggs Cape Florida State Park, suggesting that iguanas could present a threat to native and endangered species of tree snails. In Bahia Honda State Park, green iguanas have consumed nickerbean, which is a host plant of the endangered Miami Blue butterfly. As is the case with other reptiles, green iguanas can also transmit the infectious bacterium Salmonella to humans through contact with water or surfaces contaminated by their feces”.

[2]

Additionally, green iguanas appear to be responsible for power, phone, and Internet outages, as they both chew through cables and stretch themselves across power lines [1]. There is also some evidence that, on occasion, they use the burrows of other threatened species, such as the Florida burrowing owl [3].

Based on these concerns, the FWC urged homeowners to “kill green iguanas on their own property whenever possible” [2]. A few weeks later, however, they had to qualify that comment. In a press release, FWC Commissioner Rodney Barreto said:

“Unfortunately, the message has been conveyed that we are asking the public to just go out there and shoot them up. This is not what we are about; this is not the ‘wild west’. If you are not capable of safely removing iguanas from your property, please seek assistance from professionals who do this for a living”.

[4]

The FWC press release was partially motivated by a surprising amount of national coverage, which only increased after a man was hunting iguanas with a pellet gun and accidentally shot the person who was maintaining his pool [5]. However, the FWC has not changed its basic position. In the FAQ portion of its site on the green iguana, it now says this:

“Green iguanas are not protected in Florida except by anti-cruelty laws and can be humanely killed on private property year-round with landowner permission. The FWC encourages removal of green iguanas from private properties by landowners. Members of the public may also remove and kill iguanas from 22 FWC-managed public lands without a license or permit under Executive Order 17-11. Captured iguanas cannot be relocated and released at other locations in Florida. Homeowners that trap iguanas on their property may be able to obtain euthanasia services from local exotic veterinarians, humane societies or animal control offices depending on the location and availability of services”.

[2]

Florida may not be the “wild west”, but for the green iguanas, there is not much difference between the revised statement and the original one.

We do not tell this story because it is unique. To the contrary, this kind of invasive species “management” is entirely ordinary, as there have been many cases in which Fish and Wildlife commissions have tried to enlist the public to “manage” invasive species. In Texas, for instance, the state recently removed the requirement that people have a hunting license before killing feral hogs, Louisiana has long offered a bounty on nutria to encourage hunters to pursue them, Illinois offers grants to Asian carp processors to encourage them to create consumer demand for these fish, Georgia has asked its anglers to kill northern snakeheads, and Pennsylvania recently told the public to kill spotted lanternflies [6,7,8,9,10]. Instead, we tell this story because we want to explore a side of it that has not received sufficient attention in animal rights theory—namely, the extent of wrongful discrimination.

Animal rights theory claims that at least some animals are *moral patients*, i.e., beings who are deserving of direct moral consideration. We acknowledge that not all animals are moral patients, as some may not even be sentient (such as bivalves). Plainly, there is debate about the ground of moral status. Perhaps the most prominent options are associated with utilitarianism and the rights view. Peter Singer [11], for instance, argues that sentience is both necessary and sufficient for being a moral patient. By contrast, Tom Regan [12] argues that being an experiencing-subject-of-a-life is sufficient for having moral rights. We assume that, given their neurological and behavioral complexity, green iguanas are both sentient and subjects-of-a-life. Studies indicate that reptiles, including iguanas, experience pain [13,14], attempt to maximize sensory pleasure [15,16], experience basic emotional states [15,16,17], and engage in the kind of social behavior that people once assumed to be unique to mammals [18]. For instance, green iguanas have emotional responses to stressful handling experiences, which indicates “emotional fever” [19]. Although few researchers have explored reptile intelligence, the studies that have been conducted indicate that reptiles have some sophisticated cognitive capacities, including the ability to solve complex problems and apply what they have learned in the future [20,21]. Given our limited knowledge of reptile mental life and the evidence that points to reptile cognitive complexity, we ought to adopt a policy that errs on the side of caution by giving green iguanas the benefit of the doubt and treating them as if they are moral patients, as Regan [12] (p. 320) suggests for the case of late-term fetuses.

Given as much, the standard story from animal rights theory is that animals are victims of *speciesism*, often defined as the “prejudice or attitude of bias in favor of the interests of members of one’s own species and against those of members of other species” [11] (p. 6). Regan [12] (p. 155) claims that the paradigm case of speciesism is when animals are denied membership in the moral community because they do not belong to the *human* species. Regan [22] (p. 47), like Singer, emphasizes that it is speciesist to ignore the interests of animals or count them as less than the interests of human beings “simply because animals do not belong to the ‘right’ species”.

Obviously enough, any anti-speciesist is going to have serious objections to the killings that we have described. Granted, it is not costless to have green iguanas around. However, that hardly shows that the FWC has given adequate reasons for aggressive lethal management. On the one hand, we have the most significant harms that sentient beings can suffer: acute pain and death. On the other, we have weak evidence that the green iguanas have negative impacts on native species and strong evidence that they are inconvenient for human beings, raising concerns about sidewalk integrity, aesthetics, disease transmission, and the like. Moreover, even if the iguanas cause serious harm to the environment, it does not follow that they are causing serious harm to the *rights-bearing parts* of the environment—those individuals who are experiencing subjects-of-a-life. So, if people insist that the iguanas be killed, they seem to be guilty of speciesism. We might surmise, then, that there is little left for animal rights theory to say about this situation. The interests of green iguanas are being discounted relative to the interests of human beings “simply because they don’t belong to the ‘right’ species”, and, as a result, they are being unjustly killed.

But on our view, neither Singer’s nor Regan’s anti-speciesist theories explain the full extent of the discrimination at play. We suspect that what is particularly objectionable about the way green iguanas are being understood and targeted is that they are being wrongfully discriminated against, even relative to other non-human animals. To see whether this intuition can be defended, we extend Deborah Hellman’s [23] discrimination ethic to argue that the classification of green iguanas as “invasive” constitutes a kind of wrongful discrimination that is not explained by traditional accounts of speciesism. Moreover, we argue that its being wrong to categorize the green iguanas as invasive is perfectly compatible with its being morally permissible to kill them—assuming that conservationists “kill equally”. It simply is not compatible with the standard that conservationists tend to employ in their decisions about who lives and who dies.

We are not the first to criticize the way that conservationists classify species as “invasive”. Mark Sagoff [24,25,26] argues against this practice based on a number of conceptual problems: the lack of a clear account of “harm to the natural environment”, the difficulty of protecting harm impacts even after settling on an account, the overlooked species-richness that invasive species can provide, and so on. Dan Perry [27] argues that conservationists are too quick to set aside other strategies that might be employed to manage invasive species, such as translocation. Evans et al. [28] make a similar point, arguing for adaptive management over programs that seek to eliminate the relevant species. David Frank [29] argues that given the value assumptions built into the concept *invasiveness*, it is reasonable for those with differing values to reject the ways in which biologists use it to justify management practices. However, this is essentially all that environmental philosophers have had to say about the use of the term “invasive” to describe “problem” species. And while we are sympathetic to these and related criticisms, they leave a central moral issue unexplored. Is it objectionable to categorize green iguanas—and the members of many other species—as invasive simply because doing so harms those animals? In our view, the answer to that question is “No.” The reason to object to this categorization is not simply that it is harmful for green iguanas to be so categorized. It is also *demeaning* in a way that deserves special attention.

## 2. Some Preliminaries

Before we begin in earnest, though, we should be clear about the meaning of the terms “native species”, “introduction”, “alien species”, and “invasive species” as they are crucial to this discussion. Here are the definitions that the US Department of Agriculture (USDA) [30] employs:

“Native species” means, with respect to a particular ecosystem, a species that, other than as a result of an introduction, historically occurred or currently occurs in that ecosystem.

“Introduction” means the intentional or unintentional escape, release, dissemination, or placement of a species into an ecosystem as a result of human activity.

“Alien species” means, with respect to a particular ecosystem, any species, including its seeds, eggs, spores, or other biological material capable of propagating that species, that is not native to that ecosystem.

“Invasive species” means an alien species whose introduction does or is likely to cause economic or environmental harm or harm to human health.

There are many questions we might ask about these definitions. For instance, *Homo sapiens* is, of course, just one species among many. What is ecologically significant about changes that humans cause as opposed to changes that non-human species cause? Also, given that ecosystems are just groups of living and non-living entities interacting in various ways, how do we draw the boundaries of ecosystems in non-arbitrary ways? Furthermore, when we talk about species “historically occurring” in a particular ecosystem, what is the relevant timeframe? At some point in history, every single species was new to an ecosystem. Why not say that every species is an alien species?

When we try to use the above definitions to determine which species count as “native” and “alien”, a lot turns on what counts as “an introduction”. Introductions are *human* introductions—that is, human beings either intentionally or unintentionally played some role in bringing the species from one ecosystem to another as opposed to, say, animals migrating “naturally”. Obviously, there are going to be lots of cases that make these definitions seem objectionably imprecise. How should we think about a case where birds take up new nesting grounds on their own accord, but would not have if their previous nesting grounds had not been polluted by, say, a distant leak in an oil tanker—or if climate change were not happening? How should we think about a case where rats migrate from one island to another via more than one method—say, both clinging to driftwood and stowing away on ships? What about cases where species are introduced, but there is a sense in which human beings are creating an entirely new ecosystem, as occurred when American cities brought squirrels into their parks in the 19th century [31]?

We raise these questions not to suggest that they cannot be answered, but to point out that they will be very difficult to answer without explicitly appealing to an anthropocentric perspective. We draw a sharp divide between humans and non-humans; we care about some timescales and not others; we think of ourselves as being responsible for some impacts and not others; etc. As Marc Bekoff [32] suggests, conservation biology is not a value-free enterprise. It presupposes certain human goals, and it attempts to achieve those against a theoretical backdrop that is itself structured, in part, by our interests. This is most obvious, of course, in the USDA’s [30] definition of “invasive species,” which stipulates that a species counts as invasive only if it “does or is likely to cause economic or environmental harm or harm to human health”. Because human goals and interests influence the ways in which conservationists categorize free-roaming animals, these classifications are subject to moral evaluation.

## 3. Hellman Equality-Based View of Wrongful Discrimination

This leads us to the fundamental moral problem with categorizing green iguanas as “invasive”. To explain our concern, let us consider the way that Hellman [23] thinks about discrimination. We cannot argue for her view in detail here, as that would involve an extended discussion of all the rival accounts of discrimination. Our aim here is more modest. We simply want to show that (a) this is an attractive theory of discrimination on independent grounds; (b) though it excludes non-human animals, it can and should be extended to include them insofar as it is compatible with an animal rights ethic; and (c) if it is, we obtain a novel explanation—not reducible to the traditional appeal to speciesism—for our intuition that there is something distinctively wrong about categorizing green iguanas as invasive.

As Hellman points out, humans are in the business of drawing distinctions, i.e., discriminating amongst different types of creatures. Following Hellman, let us define discrimination as: drawing distinctions between creatures on the basis of some trait they possess or lack. Sometimes, of course, drawing distinctions among people on the basis of their attributes is important or unavoidable, so it follows that some instances of discrimination are permissible. Our initial concern, then, is to identify *wrongful* instances of discrimination and provide an account of why they are wrong.

We are interested in developing an ethic of discrimination that can address morally suspect instances of discrimination among non-human animals that are not adequately addressed by animal rights theory. As mentioned, traditional animal rights theory does not directly address inter-non-human-species discrimination, and it is not designed to address cases where species membership is actually relevant—as it is in wildlife management contexts, where the fact that an individual is a member of a species often *does* explain why she poses a threat to members of another species. We draw on Hellman’s plausible human-rights based discrimination ethic, as her rights-based approach is likely to be compatible with an animal *rights* framework. In doing so, we accept Hellman’s claim that discrimination is wrongful when it *demeans*, whereby to demean someone is “not merely to insult but also to put down, to diminish, and denigrate”, and “to do so in a way that has the power or capacity to put the other down” [23] (pp. 8, 29).

Hellman’s account of wrongful discrimination stems from a “bedrock moral principle”: the equal moral worth of all persons [23] (p. 6). This principle contains two sub-principles: (1) all persons have inherent value, and this requires that we treat each other with respect, and (2) the moral worth of persons does not vary according to one’s traits and, thus, does not come in degrees [23] (p. 6). The bedrock principle of equal moral worth, then, demands that we acknowledge that all people are: (1) entitled to respect and (2) equally worthy [23] (p. 6). In addition, there are two distinct ways we might violate the bedrock moral principle of the equal moral worth of persons: (1) we could fail to respect their inherent value or (2) we could fail to treat them as moral equals. This suggests that the ideas of inherent value and equal worth can come apart [23] (pp. 19, 170).

To see how inherent value and equal worth can come apart, Hellman [23] (p. 18) notes that there are things we can do to persons that would violate or deny their inherent value that have “nothing to do with a concern for equality”. For instance, “[i]f X kills Y, he fails to respect her inherent worth—but there is not an equality issue here” [23] (p. 18). While this case involves a clear violation of Y’s right to life, there are other situations in which the only right at issue is the right to be treated as an equal [23] (p. 19). For instance, someone might not have a right to rent a certain apartment, in the sense that he has no legitimate complaint against the landlord if the landlord rents it to someone else; that loss does not offend the potential renter’s inherent value [23]. After all, to fail to respect the inherent worth of another is to treat them like a mere means, tool, or resource, and when a landlord refuses to rent to a person, that person is not treated like a mere means, tool, or resource. But this does not mean that the person has not been wronged. After all, his *equal* worth can still be offended by racist selection criteria—if, for instance, the apartment complex rents only to whites [23] (p. 19). By drawing a distinction between white and Black applicants, the landlord demeans potential Black renters by expressing that they have less moral worth than white people.

Another example. Suppose that X is a thief, but he only robs women. While X certainly fails to respect the inherent value of the women he robs, insofar as he treats them as mere means to his ends, he wrongs the women in another way too: he fails to treat them as equally worthy, as he targets women exclusively. Persons, then, have not only inherent value, but also *equal moral worth*.

A benefit of Hellman’s account, then, is that it can account for wrongful differentiation that does not involve the violation of standard rights, such as the right to bodily integrity or property rights [23] (p. 10). While many assume that a commitment to the equal moral worth of persons entails that people are owed, as a matter of justice, something like freedom, resources, welfare, or capabilities, Hellman [23] (pp. 170–172) emphasizes that the equal moral worth of persons *also* commits us to treating one another as equally worthy. As she puts it, “the fact that we share a common humanity requires that we be treated *as worthy as* others” [23] (pp. 47–48).

Central to Hellman’s account of wrongful discrimination, then, are the ideas of “equality” and “equal worth”, in addition to the ideas of “respect” and “inherent value,” which are the concepts fundamental to animal rights theory. In particular, Hellman proposes that wrongful discrimination is concerned with the norms of *equality*. When you draw distinctions that demean, you fail to treat persons as moral equals, and this constitutes wrongful discrimination.

But when does discrimination demean? Hellman [23] (p. 32) suggests that whether discrimination demeans is often determined by the meaning of the discrimination in that context, as fixed by its historical and current social significance. As she points out, “our common history and culture and its conventions and social understandings… determine which actions express a rejection of the equal humanity of others” [23] (p. 35). For instance, there is arguably a moral difference between being denied a job because your last name begins with an A and being denied a job because you are a woman [23]. When someone draws distinctions on the basis of attributes that define a group that has been mistreated in the past or currently has some kind of diminished status, there is a high probability that the distinction demeans. This is because “when laws and policies group people on the basis of some [“minority”] traits, sometimes the context and culture invest these distinctions with a meaning that other distinctions do not have” [23] (p. 25).

Because our distinctions are often symbolically loaded, we must take seriously the social nature of discrimination, as what it means to express deep disrespect, to some extent, depends on culture, context, convention, and traditions [23]. Cultural meanings are attached to certain traits, such as race and sex, and “classifications made on the basis of these traits carries with [them] baggage of social significance, or association with other traits that are deeply derogatory” [23] (p. 40). Thus, the history of mistreatment and the current social status of people with a particular characteristic are relevant to determining whether instances of discrimination *on the basis of that characteristic* are wrong (i.e., demeaning).

We are not implying that it is easy to determine the meaning of a particular instance of discrimination. Sometimes, historical and present-day meanings diverge in significant ways. In other cases, a culture contains competing respect norms that leave the meaning of an act underdetermined. Still, it seems plausible that there are *some* cases where we can indeed determine the meaning of a particular act of discrimination. As we argue, this is true of the case with which we began.

To recap: Hellman proposes that the fundamental wrong-making feature of wrongful classifications is explained in terms of what you express or what you do when you draw distinctions. What you express is largely determined by convention, and conventions sometimes invest distinctions with a meaning that demeans. If you demean when you discriminate, then that discrimination is wrong. Consequently, discrimination can be wrong even if no one feels demeaned, stigmatized, or degraded, and it can be wrong even if the discriminator does not act with bad intentions [23] (p. 27).

## 4. Extending Hellman’s Account of Discrimination

Given that Hellman focuses exclusively on humanity in her discrimination ethic, we assume that she does not endorse Singer’s [33] claim that “all animals are equal”. Hellman, it seems, is guilty of wrongful discrimination insofar as, in determining who is protected by the norm of equality, she implicitly draws a distinction between “humans” and “non-human animals” that demeans non-human animals. That is, Hellman’s implicit distinction between “humans” and “non-humans” denies the equal moral worth of animals because, given the historical context of speciesist discrimination, it expresses the idea that humans, and not non-human animals, are worthy of serious moral concern. Surely, if someone were to build an anti-discrimination ethic around the principle of “the morally equality of white people”, this would demean Black people. Although this principle does not logically entail that Black people have lower moral status than white people, the principle nevertheless draws a distinction between white people and non-white people. Given the historical and current social context of racial prejudice, this discrimination expresses that Black people are worth less than white people, thereby *demeaning* Blacks.

In defending her anthropocentric theory of wrongful discrimination, Hellman might make the claim that there is a morally relevant difference between humans and non-human animals that justifies the distinction she has implicitly drawn between these groups. Here, we simply take for granted that the argument from species overlap—often referred to as the “argument from marginal cases”—provides sufficient reason to reject such a position. As many have argued, there does not appear to be any morally significant characteristic that all humans possess and all non-humans lack. Thus, we have no moral basis for granting special moral protection to a being just because she is a member of the species *Homo sapiens*. To do so would be speciesist, and speciesism is no less problematic than racism, sexism, and other oppressive “isms”.

Once we come to accept that there is no morally relevant difference between human and non-human animals, we are in a position to extend Hellman’s account of wrongful discrimination so that it goes beyond the equality of “persons” and encompasses a notion of equality for animals, human and non-human. In addition, Hellman’s discrimination ethic, when extended to non-human animals, seems to be compatible with animal rights theory, which recognizes the equal *inherent value* of animals.

According to Regan [12], the concept of inherent value is a *categorical* notion—it does not vary on the basis of an individual’s traits. Thus, humans do not have more inherent value than animals, even though rational humans can, while animals cannot, do poetry and math. Likewise, a professional physicist does not have more inherent value than the average person, even though a professional physicist can, while the average person cannot, do advanced physics. Yet, while Regan stresses that animals have equal inherent value, he does not explicitly make the further claim that animals have *equal moral worth*, and, as mentioned earlier, the ideas of “inherent value” and “equal moral worth” can come apart. Consider the following two cases:

Sarah enslaves Joe and uses him as a mere means to her ends.

Maria encounters Sam and Jake, who are both starving to death. Sarah has two meals with her. Because she thinks Sam is more attractive, she gives both meals to Sam, leaving Jake to starve to death.

Sarah treats Joe like a mere means to her ends, thereby failing to recognize his inherent value. Yet because Maria does not use Jake as a mere means to her ends, she does not deny his inherent value. But surely, she has still done something wrong. We can explain the wrongness of her behavior not by drawing on the notion of inherent value, but by drawing on the notion of *equal worth*. Maria acts wrongly because she fails to treat Sam and Jake as *equally worthy*. Assuming we should accept the norm of equality, we should acknowledge that morality requires that we refrain from drawing distinctions that demean animals, as such distinctions deny the equal moral worth of animals.

## 5. Applying the Account

If we are prepared to accept this extended version of Hellman’s discrimination ethic, then we just need to show the following: when we categorize green iguanas as invasive, we fail to treat them as equally worthy relative to other animals. To make this case, we need to get clear on what makes a classification constitute wrongful discrimination—i.e., what makes it demeaning. As discussed, Hellman suggests that we assess this by looking at the larger social context in which the discrimination occurs, the identity of the speaker, and the content of the language that is used. As we argue, the content of the term “invasive” and the context in which it occurs, certainly appear to be demeaning. But crucially, this is confirmed by the position of power that conservationists enjoy.

### 5.1. Content and Context

It is fairly clear that the term “invasive” demeans. In general, conservation biologists do not value animals as equals, and that is perhaps most obvious in the way that they typically see individual animals as expendable for the sake of species preservation. However, to categorize animals as invasive, means, for all practical purposes, “worthy of extermination”. To call iguanas invasive, then, is tantamount to saying that their kind is not worthy of existence (at least within a given region).

Roughly the same is true of categorizing the iguanas as “alien”. Consider why so many people object to describing undocumented immigrants as “illegal aliens”. Such a characterization strongly implies that these individuals are not like “us” in any of the relevant ways, do not belong in the country, and do not deserve the same sort of consideration that we give even to non-migrant foreigners. We should only expect more dramatic implications when similar language is applied to non-human animals, given the way that they are already devalued by so much of the population. By categorizing species as alien, we express that they are not valuable and are worthy of extermination.

There is a history of using genocidal practices to eliminate creatures who are classified as “invasive”. For instance, after the population of “invasive” cane toads exploded in Australia, members of the public engaged in “cane toad management” by playing “cane toad sports”, such as cane toad golf and cane toad cricket—both of which used cane toads as balls. In fact, a Northern Territory Member of Parliament encouraged the public to attack the cane toads with cricket bats and golf clubs.

Obviously, there is speciesism at work here. But it is crucial to see that speciesism does not exhaust the issue: cane toads are not just undervalued relative to human beings; they are also undervalued relative to non-human animals who are not members of “invasive” species. Both layers of discrimination are objectionable, but animal rights theory has not paid as much attention to the second. Some animals are demeaned, even relative to other non-human animals.

Moreover, while speciesism refers to the discounting of the *interests* of animals solely based on their species membership, our concern is not just that the interests of the green iguanas are discounted. After all, green iguanas do not have a preference regarding how they are categorized by wildlife managers. But still, we are concerned that the mere categorization of green iguanas as “invasive” is wrongful, insofar as the categorization puts some animals down—diminishes them—relative to other animals who are not members of species labelled “invasive”. Our claim is that it puts green iguanas down, in part, because of the historical and current treatment of animals deemed “invasive,” which involves systematic killing, often in terrible ways. Given this invasive-abusing context, drawing distinctions between “invasive” and “non-invasive” has a demeaning meaning that other wildlife management distinctions do not have, such as the distinction between “introduced species” and “native species”. It is a category that has been historically, and is still currently, used to justify exterminating species (or, at least, trying to), and, for that reason, its use indicates that the members of the species in question are less valuable than other “noninvasive” animals.

Someone might object that the real problem with the term “invasive” is that it puts animals at risk of serious harm. Granted, this is a very bad consequence of being so categorized. But our claim is that even if there were not such a risk in a particular case, the term would still demean. Again, the history of using the term “invasive” to justify harm imposition is relevant to the meaning it has when applied to any individual, at risk of harm or not, in much the way that racial slurs can demean even in cases when their use does not put anyone at risk. In short, we need to separate the consequentialist objection to categorizing animals as “invasive” from the rights-based one. When we focus on the latter, one central question concerns how animals are being viewed, and given the history of the term “invasive”, where its application is used to ”justify” mass killing, “invasive” species” are being viewed as less valuable than non-human animals generally. They are being demeaned.

### 5.2. The Power of Conservationists

Note that all this is especially objectionable, because the categorization of green iguanas as invasive is highly public and putatively authoritative. We are not the only ones to think that the former consideration affects the seriousness of degradation. Consider, for instance, what Judith Hill says:

“...an agent who treats his victim as something less than a person in public places, for the whole world to observe, demonstrates a conviction that her worthlessness is so extreme that all the world can be counted upon to regard him as justified in treating her accordingly. In short, the more public the display of contempt, the stronger is the imputation of moral worthlessness”.

[34] (p. 42)

Relatedly, we might think that part of what is so objectionable about the case of the green iguanas is the idea that a government agency can simply say that we ought to kill green iguanas on sight based on their being “invasive”, with every reason to think that their intended audience will accept their justification as entirely adequate. Green iguanas matter so little that “all the world can be counted upon to regard [the FWC] as justified in treating [the green iguanas] accordingly”.

However, the publicity is not the only factor that matters. Additionally, it is significant that that the FWC speaks with a certain sort of authority. The FWC speaks both for the state and the scientific community, and, as a result, its pronouncements have a special force. The FWC can, more or less by fiat, make it reasonable for ordinary citizens to think that they ought to kill green iguanas whenever possible; and with that power, they can affect the moral situation of ordinary citizens—they can make it excusable to take the lives of these animals. Arguably, they cannot actually make it permissible: they cannot cancel the rights that green iguanas have, as such rights are not the product of social conventions. They can, however, influence the epistemic situation of ordinary citizens such that it can be perfectly reasonable for them to think that they act as responsible citizens when they kill animals who modestly set back human interests. That kind of power makes the FWC’s use of the term “invasive” particularly heinous, as it creates an environment in which people can be more or less blamelessly recruited into very serious wrongdoing. The FWC is creating an environment that is hostile to being morally concerned for animals, and in a way that is particularly destructive for the members of one species. They are severing the normal link between wrongdoing and culpability, which damages our moral culture in a way that gives green iguanas special cause for complaint.

Obviously, green iguanas cannot recognize this state of affairs for what it is—much less complain about it. Thus, our claim is not that iguanas are somehow injured *directly* by this unequal treatment. Of course, that sort of thing certainly happens; consider, for instance, how members of certain racialized groups are injured directly when they recognize the unfairness of a moral culture that provides people with excuses for acting wrongly toward them. Non-human animals are not the victims of this kind of harm. But the point we are making is not about such harm. Instead, it is about a particularly serious kind of inequality—one that does not depend on the victim’s appreciation for its reality.

### 5.3. Discrimination in the Conservation Movement

The green iguanas are not, of course, the only victims of inequality. Insofar as many wild animals have a right to our aid—given the many ways in which we have harmed them—other “conservation” categorization schemes are problematic too. Recall that conservationists are especially active in campaigning against the killing of *iconic* animals, such as tigers, gorillas, pandas, elephants, rhinos, and polar bears. As Small [35] (p. 232) points out, conservation organizations, as well as the public at large, are “extremely sympathetic to a select number of well-known and admired species, variously called flagship, charismatic, iconic, emblematic, marquee and poster species”. While charismatic megafauna receive a considerable amount of attention from conservationists, the majority of species in need of protection are often ignored. Hugh Possingham [36] observes that, while there are approximately 20,000 species in urgent need of protection, only 80 species—commonly referred to as “celebrity species”—are recipients of conservation efforts or resources. As a result, species that are less attractive receive little attention from conservation groups.

Conservationists, then, are guilty of wrongful discrimination when they make it a priority to preserve “charismatic megafauna” while failing to dedicate similar energy, time, and resources to preserving less charismatic animals. Essentially, conservationists who grant greater protection to “charismatic” species express that not all animals are equally valuable, thereby violating the bedrock moral principle of the equal worth of animals. In choosing to first and foremost protect those “spectacular” species, they may neither violate the inherent worth of less charismatic animals, nor infringe upon the individual autonomy of less charismatic non-human animals. Yet, the *equal worth* of animals may still be “offended by the selection criteria used” [23] (p. 19)—namely, the selection criteria when deciding which species to prioritize in conservation efforts.

To defend the decision to prioritize charismatic megafauna, conservationists often argue that charismatic megafaunas are *flagship species*: “popular, charismatic species that serve as symbols and rallying points to stimulate conservation awareness and action” [37]. For instance, Sybille Klenzendorf [38], the director of World Wildlife Fund’s species program, claims that focusing on flagship species is an effective conservation tactic, as “[t]hese large, charismatic species are... the ones that require the largest amount of wild habitat, and by preserving them we save the less impressive species too”. Surely, in order to protect tigers, we must protect tiger habitat and ensure that prey animals are protected. The claim, then, is that by protecting flagship species, an umbrella effect will follow, whereby less spectacular species, as well as entire landscapes, will also be preserved. Flagship species, then, are assumed to be umbrella species: a species whose habit needs are extensive enough such that, in the effort to set aside enough space for them, conservationists simultaneously create enough habit for many other species.

But as prominent biologist Daniel Simberloff [39] (p. 247) argues, “whether many other species will really fall under the umbrella is a matter of faith rather than research.” As he claims, a flagship is not necessarily a good indicator or umbrella species, since there is not always biodiversity (for instance, diversity of invertebrates, reptiles, or amphibians) in the habitats of charismatic megafauna [39]. Likewise, Williams et al. [40] conclude that areas where the “Big Five” African mammals (lions, leopards, elephants, African buffalo, and rhinos) are found do not have a higher rate of biodiversity of other species than random areas of land. Small [35] (p. 232) substantiates these reports, finding that “there is only limited evidence of ‘trickle-down’ benefits to rare, endemic and endangered species.” The moral of the story is that when conservationists focus exclusively on preserving select charismatic species, they fail to promote overall biodiversity. Thus, conservationists cannot justify prioritizing “flagship species” by appealing to an “umbrella effect”.

If conservationists were to take our discrimination ethic seriously, they would come to see that it is wrong to draw distinctions that express the anti-egalitarian message that some animals are worth less than others. The problem with the conservation movement is not simply that conservationists fail to treat animals with respect or that they treat animals as if they lacked inherent worth, though those accusations are often fair. Additionally, the conservation movement has policies and practices that wrongfully discriminate, insofar as they express that some animals have less moral worth than others. Still, we might think that failures to aid are often less problematic than actively causing harm. So, it remains the case that there is a particular grievous wrong being done to green iguanas—and members of similar species—who are categorized in ways that make them targets.

## 6. Animal Rights Theory and Killing

Our aim was to explain how animal rights theory can and should register a particular moral complaint against the practice of categorizing some animals as “invasive”. Someone might worry, however, that despite all we have said, the real issue is the killing, and discrimination is only objectionable when it results in the systematic culling of particular animals. In closing, then, we want to stress that the issue of killing is, in fact, separate from the issue of discrimination. We can see this most easily by reflecting on the conditions under which animal rights theory actually sanctions killing—namely, when the aim is to protect rights and discrimination is carefully avoided.

Regan’s [12] animal rights theory provides us with two principles for handling cases where there are conflicts between rights: the “miniride” principle and the “worse-off” principle. Miniride says, in essence, that we should minimize the number of individuals whose rights are overridden when we are considering comparable rights violations:

“[W]hen we must choose between overriding the rights of many who are innocent or the rights of few who are innocent, and when each affected individual will be harmed in a prima facie comparable way, then we ought to choose to override the rights of the few in preference to overriding the rights of the many”.

[12] (p. 305)

This principle makes it possible to generate cases where it would be perfectly appropriate to kill green iguanas. Suppose—contrary to fact—that the following were true:

Green iguanas prey on a particular tree frog. The tree frog is itself an “alien” species, introduced in the same way that green iguanas were, namely, by being abandoned by former owners who originally imported them as pets. The tree frog is not the only food that iguanas can eat; they just happen to have a strong preference for the tree frogs over the plant and insect options available to them. Any given iguana will eat thousands of tree frogs over the course of her lifetime; as a result, iguanas are, collectively, bringing the tree frog population down to a critical level. We have no way to translocate the members of either species; reproductive management is not possible. The only feasible way to spare tree frogs from predation is to kill the iguanas.

In such circumstances, it may be true that we must choose between overriding the rights of many who are innocent (the tree frogs, who we have placed in harm’s way, and so to whom we have a duty of aid) or the rights of the few who are innocent (the green iguanas, who are innocent threats). Moreover, each affected individual will be harmed in a prima facie comparable way, namely, by dying. So, according to Regan’s miniride principle, we ought to choose to override the rights of the few rather than override the rights of the many. But the reason for choosing to kill iguanas over the frogs does not express the belief that iguanas have less moral worth than tree frogs. We are just trying to minimize the number of comparable rights violations.

Regan himself, then, implicitly acknowledges that in forced-choice situations, our decisions about who lives and who dies ought to respect norms of equality. You receive no extra consideration simply because you are a human or a charismatic animal. Thus, when faced with difficult moral dilemmas that involve prima facie comparable harms, we ought not to draw distinctions on the basis of arbitrary characteristics, such as race, species, intelligence, or charisma. Rather, we ought to make these difficult life and death decisions *only* by looking at the numbers. In doing so, we avoid demeaning those who already get the raw end of the deal.

Conservationists may object that this is wildly idealistic. Given limited conservation budgets, non-lethal strategies are often unavailable. In addition, if lethal strategies are forbidden, then conservationists will have their hands tied. Species will be lost and ecosystems will change.

No one has ever claimed that animal rights theory fits neatly with traditional conservation goals. We grant that it would be massively more difficult for wildlife managers to achieve their aims in a rights-respecting way. To some degree, that is a way of condemning those aims. However, it is worth remembering that we are still in the early days of animal rights theory. The core framework is only a few decades old, and it has mostly been used to criticize common practices—not in seeking to reform them. It would be good to see more work that tries to defend some range of conservation projects in a rights-respecting way. If there are surprising points of agreement between conservationists and animal rights theorists, then that may make it slightly easier to convince conservationists that animal rights theory is worth taking seriously. If not, then the battle lines will be clearer.

## 7. Conclusions

For the foreseeable future, we will be called upon to make painful decisions about which animals live and which animals die. In many cases, the right decision will not be obvious, as we are not always confident about the effects of intervening in ecosystems. But we can be confident that we have made at least one morally sound decision if we refuse to draw distinctions among animals that categorize some, but not others, as “invasive”. In other words, we can be confident that we have made at least one morally sound conservation decision when we refuse to draw distinctions that demean.
